# The risk for major depression and bipolar disorder in the offspring of informative parental mating types: a Swedish population-based study

**DOI:** 10.1017/S0033291726103286

**Published:** 2026-02-11

**Authors:** Kenneth Kendler, Jan Sundquist, Kristina Sundquist, Linda Abrahamsson

**Affiliations:** 1 Department of Psychiatry, Virginia Commonwealth University, Richmond, USA; 2 Lund University: Lunds Universitet, Lund, Sweden

**Keywords:** bipolar disorder, dual mating study, environmental risk score, major depression, stressful life events

## Abstract

**Background:**

Seeking to clarify the parent-offspring transmission of Major Depression (MD) and type I Bipolar Disorder (BD), we examined offspring MD and BD risk in five informative parental pairs: Unaffected x MD, Unaffected x BD, MDxMD, MDxBD and BDxBD.

**Methods:**

We identified 289,637 individuals born in Sweden 1970-1990, followed through 2018, from parents with MD and/or BD identified from Swedish medical registers. We quantified the MD→MD, BD→BD, MD→BD and BD→MD parent-offspring transmission and explored effects of parental illness on MD→BD conversions.

**Results:**

The risk for MD was modestly and similarly increased in offspring of Unaffected x MD (HR=1.64) and Unaffected x BD parents (HR=1.53), higher in MDxMD and MDxBD pairings (HRs=2.39 and 2.47) and slightly lower in BDxBD matings (HR=2.29). By contrast, risk for BD was much higher in Unaffected x BD versus Unaffected x MD matings (HRs = 5.59 vs. 1.70), further elevated modestly in MDxBD matings (HR=6.26) and very high in BDxBD matings (HR=13.61). The rate of offspring MD→BD conversions was substantially increased by parental BD but not parental MD. Offspring BD was equally predicted by paternal and maternal affective illness while offspring MD was more strongly predicted by maternal than paternal affective illness.

**Conclusions:**

Examining risk for MD and BD in offspring of different parental mating types of MD and BD is an informative strategy for further clarifying the cross-generational transmission of these two partially related and partially distinct mood disorders.

The debate about the etiologic relationship between depression and mania goes back well into the 19th century. Our modern concept of bipolar illness was first suggested by Falret (1854) and Baillarger (1854). Kraepelin (1899), in his 6th edition textbook, first articulated his synthetic and expansive view of mood disorders nearly all forms of which were incorporated into his category of manic-depressive insanity (MDI). In the decades after this text, Kraepelin was repeatedly criticized for the breath and heterogeneity of this syndrome (Kendler & Engstrom, 2018). The major 20th challenge to Kraepelin’s concept of MDI came later from Leonard (1959), who, in his 1959 textbook, argued that Kraepelin’s categories of major depression (MD) and bipolar disorder (BD) should not be considered part of the same diagnosis but were rather distinct disorders. Family studies of BD and MD, especially those conducted by Perris (1966) and Angst (1966) showed distinct patterns of aggregation and co-aggregation for MD and BD and played an important role in the acceptance of the MD-BD difference. This distinction was recently formally recognized by the APA Diagnostic and Statistical Manuel which in DSM-IV (1994) had an overall category of Mood Disorders while in DSM-5 (2013), separate categories were given to ‘Depressive Disorders’ and ‘Bipolar and Related Disorders’.

As indicated by several reviews of the relatively large number of family studies examining risk of MD and BD in first-degree relatives of MD versus BD probands (Rasic et al., 2014; Smoller & Finn, 2003; Tsuang & Faraone, 1990; Uher et al., 2023; Wilde et al., 2014), the patterns of results, found across most, but not all studies, suggest two major findings. First, risk for BD is substantially elevated in relatives of BD probands while the excess risk for BD in relatives of MD probands is much more modest. Second, risk for MD is moderately elevated in relatives of both MD and BD probands to a broadly similar extent. These results are typically interpreted as suggesting that MD and BD are related conditions but not, from a familial perspective, sufficiently similar to be considered subtypes of a single disorder.

This literature has several potential limitations. First, sample sizes of relatives were often limited, resulting in imprecise estimates especially for BD, a relatively rare syndrome. Second, definitions of BD varied and recently there were differences of opinion about whether BD II cases were included. Third, the age of the MD probands in such studies were often in early to mid-adult life so they had not completed their age at risk for conversion to BD (Kessing et al., 2017). Fourth, representativeness of cases varied with some studies ascertaining cases only at highly specialized research facilities (Gershon et al., 1982) and others in out-patient clinics (Weissman et al., 1984). Fifth, conversion of MD to BD cases in relatives was not, to our knowledge systematically examined in any of the studies although it is informative about familial transmission of these two syndromes. Finally, to our knowledge, all these studies identified affected cases and controls and then studied their first-degree relatives. None of these studies examined risks in offspring of the five possible mating types of parental MD and parental BD compared to unaffected (UN) x UN matings (termed type A) if sex of parent is not considered: B) MD x UN, C) BD x UN, D) MD x MD, E) MD x BD, and F) BD x BD. Being able to compare risks across these mating types allows for a richer analysis than is possible from probands with only MD or BD. In particular, they allow an analysis of how risk changes when one versus both parents have MD or BD and how having a coparent with MD changes the risk in offspring of a BD parent and a coparent with BD changes the risk in offspring of an MD parent.

We therefore here examine, using the resources of the Swedish national registers, the parent-offspring transmission of the risk for MD and BD and for MD to BD conversion from the five possible mating types. We then examine differences in maternal versus paternal risk transmission, another methodologic advantage of using parent–offspring family designs.

## Methods

Swedish population-based medical registers and a nearly nationwide primary health care research database, were used for finding information on study individuals (Appendix Table 1). Data sources were linked by using a unique identification number of each individual, replaced with a serial number by Statistics Sweden for confidentiality. Ethical approval was secured from the regional ethical review board in Lund, Sweden. We identified all individuals born in Sweden 1970-1990, with available follow-up time at least until age of 15 (*n* = 2,071,848). Follow-up data were available until December 31, 2018. By the use of the multigeneration register, each individual was linked to his/her biological parents. To be included in the study, the individuals had to be raised in an intact family (living in the same household as the biological mother and father between ages 0-15), excluding triads in which at least one of the offspring, mother, or father were diagnosed with schizophrenia (SZ), leading to a sample size of 1,237,306. For defining intact families, we made use of household identification numbers from the Population and Housing Census, updated every fifth year, between the years 1970 to 1985. For years with no information on whether offspring and parents were registered at the same residence, we made use of data from the closest year. From 1986 onward, we used yearly information on the family identification from the Total Population Register, defined as individuals registered at the same property who are related, married, or have common children.

Diagnoses of MD, BD (excluding ICD10 codes of F30.0 and F31.0: hypomania and hypomanic episodes), and SZ in offspring and parents were searched for using the Swedish Hospital Discharge Register, Outpatient Care Register and primary care data (for details, see Appendix Table 2). Thus, we were, in this sample, cases whose only BD diagnosis involved hypomania were excluded from our BD diagnostic category. Our intent was to only study classical or BD type I.

Three outcomes were defined in the study: i) BD total (having at least one BD registration), ii) MD only (having at least one MD registration but no BD registration), and iii) BD conversion (having at least one BD registration, amongst those with a prior MD registration and at least 5 years of follow-up time available, following the MD onset, *n* = 116,405).

In intact families, we studied the effect of lifetime parental diagnoses of MD and BD on the three different outcomes i–iii in offspring. We made use of two complimentary statistical measures that both provide measures of parent–offspring diagnostic similarity: tetrachoric correlations (TC) and hazard ratios (HRs). TCs have the advantages of being easy to interpret (Falconer, 1989) and base rate insensitive. (Babchishin & Helmus, 2016) thus being a valuable measure when comparing parent-offspring resemblance for disorders with quite different prevalences, such as MD and BD. They are also easy to interpret in a genetic-epidemiological framework as they reflect the correlation in the familial liability between relatives (Falconer, 1960). HRs, on the other hand, are sensitive to base rates, but have the advantage over TCs that, via use of time to event regression modelling, it is a built-in feature in statistical programs to correct for follow-up time and confounding. HRs are also used more commonly in both family and epidemiological studies than are TCs.

Within the intact families, parental sex-neutral dual mating types were categorized into six different groups. In doing so, we start with defining a UN parent as a parent without any MD or BD diagnosis, whilst an MD^+^ parent was diagnosed with MD only and an MD^-^ parent did not have MD only, etc. As for offspring, we did not take into account whether parents were having both MD and BD, in such cases only the BD diagnosis was counted. Labelled A-F, following are the six groups: A: UN x UN, B: BD^−^MD^+^ x UN, C: BD^+^MD^−^ x UN, D: BD^−^MD^+^ x BD^−^MD^+^, E: BD^−^MD^+^ x BD^+^MD^−^, and F: BD^+^MD^−^ x BD^+^MD^−^. A total of 289,637 offspring (23.4%) had at least one parent diagnosed with MD or BD.

Firstly, tetrachoric correlations (TCs), which estimates the correlation between two underlying continuous latent variables from binary data, were calculated using the ci.tetra function in the statpsych R package (Bonett & Calin-Jageman, 2025), which is based on methodology presented by Bonett and Price (2005). The methodology, which builds on an odds-ratio approximation, has been further extended, (Bonett & Price, 2007) and has been found to perform well compared to other methods (Savalei et al., 2015). It has also been applied extensively (Esberg et al., 2020; Milosevich et al., 2024; Nambiema et al., 2023). TCs were calculated, comparing the offspring outcome of interest dependent on parental dual mating type. When for example wanting to compare parental groups B and A, the TC was retrieved from a two-by-two table, with being exposed to parental group B vs. A on one dimension, and having the offspring outcome or not on the other.

Secondly, hazard ratios were retrieved from Cox proportional hazards regression, in which time to first diagnosis of MD or BD in offspring from the age of 10 (or from age at onset of MD for BD conversion) using a multivariable Cox proportional hazards model with age of offspring (time since MD onset for BD conversion) as the time scale. The offspring were followed until diagnosis, end of study (December 31, 2018), death, or emigration – whichever occurred first. In all models, we control for sex and year of birth of offspring and year of births of the mother and father. In the BD conversion model, we also corrected for age at MD onset. To remove problems with nonproportional hazards in the models based on age as time scale, we include interaction effects between age of offspring and controlling variables by using a procedure of splitting data over time (Zhang et al., 2018). The different sex-neutral parental dual mating groups were included as dummy variables.

To further estimate sex-specific parental effects, firstly for TCs, in order to, for example, retrieve an estimate of mother’s BD, we compare parental group A to families in which the mother is having BD, whilst the father is not having any type of diagnosis. Secondly, for HRs, we remove the variables of dual parental diagnostic combinations to instead include four main effects variables of mother’s BD, mother’s MD, father’s BD, and father’s MD. Further, for testing whether e.g. the effect size of mother’s BD equaled that of father’s BD, we included also all possible interaction effects between the four main effects (removing the impact from parental groups with more than one diagnosis), and contrasted the difference between the two effect measures.

Levels of significance 0.05, 0.01, 0.001, and 0.0001 were marked in tables. Data analysis was conducted from March 11, 2025, to September 23, 2025. Statistical analyses were performed using R, version 4.4.2 (R Core Team, 2024) (Appendix Table 3) and SAS, version 9.4 (2016).

## Results

Table 1 provides descriptive statistics of the sample of 1,237,306 offspring of intact mother-father parental pairs that we examine here. At the time of last follow-up, parents were in their mid-to-late 60s, having lived through nearly all of their risk for MD and BD and the risk for MD → BD conversions. Their children were, on average, in the late 30s. In the children’s generation, who lived at time when the registries were largely complete and functioning well, the mean age at onset of both MD and BD were in the early 30s and the lifetime prevalence for MD and BD were 14.3 and 1.1% respectively. In parents who had completed their ages at risk for MD and BD, the lifetime prevalence of MD was much higher than BD, with both mood disorders more common in mothers than fathers. A similar pattern was seen in sons and daughters. In sons and daughters with a first onset of MD and a minimum of 5 years of follow up, the rate of conversion to BD was 6.9%.

**Table 1.** Descriptive statistics of the study in terms of sample size, birth year, age, sex distributions, parental and offspring prevalences, conversion rates of MD → BD and ages at first registration of bipolar disorder (BD), and major depression (MD)

	All offspring		Female offspring		Male offspring		Biological mother		Biological father	
Number	1,237,306		595,695		641,611		1,237,306		1,237,306	
	*M*	*SD*	*M*	*SD*	*M*	*SD*	*M*	*SD*	*M*	*SD*
Year of birth	1979.9	6.1	1979.9	6.1	1979.8	6.1	1951.7	7.1	1949.0	7.5
Age at follow-up	37.7	6.8	37.5	6.9	37.8	6.8	66.2	7.2	68.3	7.3
**Prevalence rates, %**										
BD total	1.13		1.47		0.81		0.85		0.65	
MD only	14.27		18.74		10.12		15.37		9.03	
**Conversion rates**										
MD -> BD^a^	6.93		7.19		6.44		4.09		4.88	
**Age at first diagnosis**										
	*M*	*SD*	*M*	*SD*	*M*	*SD*	*M*	*SD*	*M*	*SD*
BD total	30.9	6.8	30.6	6.7	31.3	6.9	51.5	11.9	54.8	11.6
MD only	31.2	7.0	30.9	7.0	31.5	7.0	56.3	10.2	59.7	10.7

a All with first MD, after total follow-up time (among those with at least 5 years of follow-up time available).

As described in the methods, we employ two complimentary statistical approaches to the quantification of parent-offspring transmission of the risk to MD and BD and rates of MD conversion to BD: tetrachoric correlations (TC) and hazard ratios (HR). Table 2 describes the TCs and HRs and 95% CIs for the six possible mating types, in columns B to F, all compared with the offspring of parents who are both unaffected for both MD and BD in column A as well as the change in rates of MD → BD conversion. The sample size of our offspring groups varies widely between mating types from over 240,000 with one parent with MD to 113 with both parents with BD. These differences in sample sizes are mirrored by differences in the size of our 95% confidence intervals.

**Table 2.** Tetrachoric correlations (TC) and hazard ratios (HR) of mating combinations of parental bipolar disorder (BD) and major depression (MD) with offspring BD and MD

Tetrachoric correlations											
	A. UN x UN	B. BD^−^**MD**^**+**^ x UN		C. **BD**^**+**^MD^−^ x UN		D. BD^−^**MD**^**+**^ x BD^−^**MD**^**+**^		E. BD^−^**MD**^**+**^ x **BD**^**+**^MD^−^		F. **BD**^**+**^MD^−^ x **BD**^**+**^MD^−^	
Number of offspring	947,669	243,878		14,971		27,340		3,335		113	
Offspring diagnosis		TC	95% CI^a^	TC	95% CI^a^	TC	95% CI^a^	TC	95% CI^a^	TC	95% CI^a^
BD total	Reference	0.15	0.14, 0.16****	0.48	0.46, 0.50****	0.29	0.27, 0.32****	0.50	0.47, 0.54****	0.68	0.58, 0.76****
MD only	Reference	0.18	0.17, 0.18****	0.13	0.12, 0.14****	0.27	0.26, 0.28****	0.27	0.25, 0.29****	0.26	0.15, 0.37****
Conversion to BD^b^	Reference	0.03	0.02, 0.05***	0.35	0.31, 0.38****	0.09	0.05, 0.12****	0.27	0.22, 0.33****	0.51	0.32, 0.66****
**Hazard ratios**											
BD total	Reference	1.70	1.64, 1.77****	5.59	5.16, 6.05****	2.74	2.53, 2.97****	6.26	5.39, 7.27****	13.61	7.24, 25.57****
MD only	Reference	1.64	1.63, 1.65****	1.53	1.50, 1.56****	2.39	2.36, 2.42****	2.47	2.39,2.55****	2.29	1.98, 2.64****
Conversion to BD^b^	Reference	1.05	1.00, 1.11	2.94	2.65, 3.26****	1.19	1.08, 1.31***	2.21	1.83, 2.67****	4.90	2.41, 9.95****

a Significance levels for *p*-values: *<0.05, **<0.01, ***<0.001, ****<0.0001.

b All with MD onset first are included, having at least 5 years of follow-up after onset; *n* = 116,405 offspring.

Focusing first on TCs in the top of Table 2, one parent with MD (Column B) produces a tetrachoric correlation (95% CIs) with the offspring of +0.18 (0.17-0.18) for MD and +0.15 (0.14-0.16) for BD and a slight but significant elevation of the conversion rate of +0.03 (0.02-0.05). The results for one parent with BD (column C) are quite different with a much higher elevation of BD risk (TC = +0.48; 0.46-0.50), and MD → BD conversion (TC = +0.35; 0.31-0.38) and a smaller increase in MD risk (TC = +0.13; 0.12-0.14). In column D, which presents results for a dual mating of parents with MD shows, in comparison with column B, substantial rises in risk for MD (TC = +0.27; 0.26-0.28), BD (TC = +0.29; 0.27-0.32) and the conversion rate (TC = +0.09; 0.05-0.12). Column E examines the offspring of MD x BD parental pairs, and can be conceptualized as a combination of the matings seen in columns B and C. The TC for BD is only slightly higher than that seen in column C (+0.50; 0.47-0.54) while the TC for MD is identical to that seen in MD x MD matings (0.27; 0.25-0.29). Interestingly, the conversion rate is lower than that seen in column C (+0.27; 0.22-0.33), but known quite imprecisely. Column F – results from the rare BD x BD matings (with the associated relatively large CIs) – shows a considerably higher risk for BD (TC = +0.68; 0.58-0.76) and for MD → BD conversion (TC = 0.51; 0.32-0.66) – but a risk for MD virtually identical to that seen in columns D and E: +0.26 (0.15-0.37).

While the results using HRs mirror closely the general trends seen using TC, some differences are noteworthy and driven by differences in the statistical methods, particularly that HRs are much more base-rate sensitive than TCs and hence for similar TCs will produce larger HRs for BD which is more than 10 times rarer than MD. For example, in column B – one parent with MD – our TC analyses show a higher risk for MD than for BD in offspring, as might be expected. However, the HR analyses show the reverse. Another example is the change in risk for BD in columns E to F where the TC presents a ratio of 0.68 to 0.50 or 1.36:1 and HRs present a much higher ratio of 13.61 to 6.26 or 2.17:1.

Our dual family model provides three different and independent estimates for the effect of parental MD on risk for MD each with a different background risk: comparing the risk in MD x UN parents with control UN x UN parents (Models B versus A, already reported in Table 2); MD x MD versus MD x UN (models D versus B) and 3) MD x BD vs BD x UN (model E versus C). Given their lack of base-rate sensitives, TCs are, we suspect, a better statistic for these comparisons. The TCs for these three comparisons equal, respectively, +0.18 (0.18-0.19), 0.13 (0.12-0.14) and +0.18 (0.15-0.21).

We then performed the same analysis for BD, which compares the following mating types: BD x UN parents with control UN x UN parents (Models C versus A, already reported in Table 2); MD x BD versus BD x UN (Models E versus B) and 3) BD x BD vs BD x UN (Models F vs C). The TCs for these three comparisons equal, respectively, +0.48 (0.46-50), 0.38 (0.34-0.42), and +0.30 (0.14-0.44).

Our prior analyses did not differentiate between mothers and fathers in the familial transmission of MD and BD which we examine in Table 3. The risk for BD from maternal versus paternal MD and from maternal or paternal BD was nearly identical using both TCs and HRs. However, risk for offspring MD from either parental MD or BD was stronger when transmitted from the mother versus the father. We tested these interaction using HRs and both were modest but statistically significant: parental versus maternal BD (HR = 1.11, 1.02-1.21) and parental versus maternal MD (HR = 1.07, 1.05-1.10).

**Table 3.** Sex-specific effects of parental bipolar disorder (BD) and major depression (MD) on the tetrachoric correlations (TC) and hazard ratios (HR) in offspring for BD and MD

Tetrachoric correlations						
Offspring diagnosis	BD total		MD only		Conversion to BD^b^	
Independent variables of parental diagnoses	TC	95% CI^a^	TC	95% CI^a^	TC	95% CI^a^
Mother’s BD	0.48	0.45, 0.50****	0.14	0.13, 0.16****	0.34	0.30, 0.38****
Mother’s MD	0.16	0.15, 0.17****	0.19	0.18, 0.19****	0.03	0.01, 0.05***
Father’s BD	0.48	0.45, 0.51****	0.11	0.07, 0.15****	0.34	0.29, 0.39
Father’s MD	0.15	0.13, 0.16****	0.15	0.15, 0.16****	0.03	0.01, 0.06*
**Hazard ratios**						
Offspring diagnosis	BD Total		MD Only		Conversion to BD^b^	
Independent variables of parental diagnoses	HR	95% CI^a^	HR	95% CI^a^	HR	95% CI^a^
Mother’s BD	5.05	4.61, 5.54****	1.58	1.51, 1.65****	2.61	2.31, 2.94****
Mother’s MD	1.70	1.63, 1.77****	1.66	1.64,1.68****	1.05	0.99, 1.10
Father’s BD	4.80	4.32, 5.34****	1.47	1.39, 1.55****	2.71	2.36, 3.11****
Father’s MD	1.60	1.53, 1.68****	1.52	1.50, 1.54****	1.08	1.01, 1.15*

a Significance levels for *p*-values: *<0.05, **<0.01, ***<0.001, ****<0.0001.

b All with MD onset first are included, having at least 5 years of follow-up after onset; *n* = 116,405 offspring.

## Discussion

With the aim of further clarifying the familial relationship between MD and BD, we studied, in the Swedish national registries, the risk for MD, BD, and MD → BD conversion in offspring in the five possible MD and BD parental mating types compared to the risks seen in offspring of two unaffected parents. We utilized two complimentary statistical approaches: tetrachoric correlations and hazard ratios.

Of the many results obtained, we emphasize seven. First, consistent with nearly all prior reviews, (Rasic et al., 2014; Smoller & Finn, 2003; Tsuang & Faraone, 1990; Wilde et al., 2014) the familial transmission of BD is stronger than is seen for MD. One parent affected with MD produced a TC and HR for MD in offspring of, respectively, +0.18 and 1.64, while for BD in one parent to BD in child, the parallel results were +0.48 and 5.59. How typical are our results of the prior literature? In interpreting our results, it is important to note that while our population prevalence estimate for BD – 1.1% – is exactly the same as that estimated in a recent meta-analysis (Clemente et al., 2015), our prevalence estimate for MD – 14.3% – is on the high end, especially for registry-based studies. For example, the mean risk for MD in controls for the five most definitive family studies of MD is 7.1% (Sullivan et al., 2000) and the largest meta-analysis of MD prevalence worldwide yielded an estimate of 6.7% (Waraich et al., 2004).

Our results for MD are in the range of a large-scale meta-analysis of father to offspring transmission of MD, estimated at OR = 1.42 (1.17-1.71) (Dachew et al., 2023) but considerably lower than those seen in a 2014 and 2023 meta-analyses of MD parent-offspring transmission: RR = 2.38 (1.94-2.71) (Rasic et al., 2014) and 2.3 (1.9-2.6), respectively (Uher et al., 2023). One of the advantages of utilizing TCs in these analyses is that genetic theory (Falconer, 1989) predicts that if environmental effects make only small contributions to parent-offspring resemblance, then the heritability of a disorder should equal approximately twice the parent–offspring TCs. Doubling the parent–child TC for MD equals +0.36, very close to the 37% represented by the best available meta-analysis of MD twin studies (Sullivan et al., 2000). This pattern of findings – somewhat lower HRs or ORs than most prior studies but nearly identical TCs is what would be expected given our relatively high prevalence rates for MD. This is because, with rising prevalence rates for the same degree of association, tetrachoric correlations are relatively stable (Olsson, 1979) while HRs decline (Hernán, 2010).

Our findings for BD → BD parent-offspring transmission are somewhat stronger than those presented by Rasic et al. in their 2014 meta-analysis (RR = 4.1 (1.9-8.6) (Rasic et al., 2014) and Uher et al. in their 2023 effort: RR=5.1 (3.3-8.1) (Uher et al., 2023) but well within their estimated CIs. This is expected given our prevalence rates for BD are quite typical of those found in other samples. Doubling the parent-child TC for BD produces an approximate heritability of 96% which is moderately higher than the 85% estimated by one of the best twin studies of BD (McGuffin et al., 2003) and higher than the range of twin heritabilities of ‘between 60% and 90%’ given by a recent review (O’Connell & Coombes, 2021).

Second, in families with one parent with MD, the MD → BD cross-generational transmission is similar in magnitude to that seen for MD → MD both as assessed by TC (+0.15 vs +0.18) and HR (1.70 vs. 1.64). These results are congruent with those presented in the Uher et al. meta-analysis of cross-generational transmission: MD → BD and RR = 2.1 (1.5-2.9) versus MD → MD and 2.3 (1.9-2.6). (Uher et al., 2023) Third, in families with one parent with BD, we see a quite different picture. By both TC and HR, the BD→BD transmission is much stronger than the BD → MD transmission (TCs +0.48 vs 0.13 and HRs 5.59 vs 1.53). These findings are again consistent with nearly all prior studies (Rasic et al., 2014; Smoller & Finn, 2003; Tsuang & Faraone, 1990; Uher et al., 2023; Wilde et al., 2014), for example, the parallel results in the Uher meta-analysis were RR=5.1 (3.3-8.1) and 2.1 (0.9-5.0) (Uher et al., 2023).

Fourth, of empirical interest, we could estimate the risks for MD and BD from parental dual-matings. For MD x MD matings, the increased risk for offspring MD was still relatively modest (TC = 0.27 and HR = 2.39. More dramatic results were seen in the offspring of the relatively BD x BD matings, however: TC = 0.68 and HR = 13.61. The most comparable study we could find for these latter results was based on Danish registries and reported a RR for BD in offspring of two versus one affected parent equal to 5.7, (Gottesman et al., 2010) considerably higher than that seen in our data (~ 2.4).

Fifth, because of our design, we can test the impact of spousal mood disorders on the MD → MD and BD → BD parent–offspring transmission. For MD → MD transmission, we found that having the other parent being unaffected or having BD produces the same risk for MD in the offspring from an MD parent. However, the risk for MD for a second parent when one parent already has MD is significantly attenuated. For BD, the pattern was more complex and suggest that, compared to having a background other parent who is unaffected, the risk for BD is modestly attenuated by having another parent with MD and more strongly attenuated when the other parent has BD. These results have implications for comprehensive models for the familial transmission of mood disorders.

Sixth, parent–offspring transmission of BD risk was virtually identical from fathers and from mothers, while for MD, modest, but significantly greater transmission was seen for mother–offspring than father–offspring pairs. The prior relatively well powered study that addressed this question (Lieb et al., 2002) found no significant difference in risk for MD from affected mothers versus fathers. However, our results are consistent with a large meta-analysis of a range of internalizing disorders showing modestly stronger transmission from mother to child versus father to child (Connell & Goodman, 2002).

Seventh, for the first time to our knowledge, we examined, in those offspring who developed MD, the effects of parental mood disorders on the risk for MD → BD conversion. Interestingly, parental MD produced a slight but statistically significant increase in that conversion rate while parental BD produced a large increase (TR = +0.35 and HR = 2.04).

We should briefly note differences between the present study and the prior 2015 detailed study of BD in Sweden by Song et al. (2015) Compared to their study, we had an additional decade of follow-up, included primary care data – crucial for our MD analyses – examined mating types and explored MD → BD conversions.

Consistent with the results of a prior detailed review of the relevant literature that included a population wide analysis in Sweden, (Rhee et al., 2023) we found that MD → BD conversion rates were higher in our female than male offspring. These results are in contrast to our findings that this rate was higher in fathers than mothers. We have no good explanation for this generational difference.

Finally, what might we conclude from our analyses regarding the vexed question of the etiologic relationship between MD and BD, as seen from a familial perspective? First, we find modest to moderate cross-aggregation – increased rates of BD in offspring of MD parents and MD in offspring of BD parents, which strongly supports a sharing of familial risks between these disorders. Second, we find much stronger BD → BD than BD → MD transmission, which suggests a considerable proportion of familial risk for BD is not shared with MD. Third, we find much less specificity in the MD → MD transmission, which suggests an asymmetry in the sharing of familial risk between the two disorders – that is that more familial MD liability is shared with BD than the other way around. Fourth, descriptively, our results are not consistent with the previously postulated multiple threshold model for BD/MD (Baron, 1980; Kendler et al., 1995), which posits a single underlying familial liability to BD and MD, with the former being a result of higher liability than the latter. For example, this model would predict a substantially larger risk for MD in offspring of BD versus MD parents, which we do not see. Thus, we can, consistent with most prior findings, reject the two extreme hypotheses that the correlation in familial risk between the disorders is very low to non-existent or very high and close to unity. Rather it is somewhere in the moderate range but with a striking asymmetry in that a much higher proportion of the familial risk for BD is unique to that disorder and not shared with MD, while for MD, the sharing of familial risk with BD is quite high.

## Limitations

These results should be considered in the context of two potentially important limitation. The diagnoses of BD and MD from the Swedish registries are not research-based but rather represent an averaging of the diagnostic approach of Swedish psychiatric clinicians over decades. However, the validity of Swedish hospital diagnoses for BD are well supported (Sellgren et al., 2011) as is the validity of MD based on its prevalence, sex ratio, sibling, and twin correlations and associations with psychosocial risk factors (Kendler et al., 2018; Sundquist et al., 2017).

Second, we included in our sample only intact families where offspring resided with both parents up to age 15. We did this to ensure that all offspring received both their genes and their rearing from their biological parents. However, an inevitable consequence of this decision was to exclude various types of ‘broken’ families. To examine what kind of biases we may have thereby introduced into our analyses, we repeated the main analyses of this paper on the broken families in our sample and present the results in Appendix Tables 4 and 5. As expected, the rates of MD and BD were consistently higher than seen in the intact families. The overall pattern of results for TCs and ORs, however, were reassuringly similar for nearly all the analyses. Quantitatively, the TCs in the intact and broken families were quite close in value while the ORs in the broken families were modestly lower than in the intact families, consistent with the greater base-rates sensitivity of the OR statistic.

## Conclusions

This study of the risk for MD and BD in offspring of affected parents has six major strengths: (i) representative sampling, (ii) large sample size, (iii) consideration of the five possible mating types, (iv) joint consideration of two different metrics of familial resembles (TCs and HR), and (v) the consideration of MD → BD conversions. The multiple mating types permit us to examine the parent–offspring transmission of risk against different backgrounds of risk in the spouse where the risk for MD → MD and BD → BD transmission attenuates modestly if the spouse has the same disorder. As might be expected, the MD → BD conversion rates in offspring are predicted much more strongly from BD than MD in parents. Our findings support a moderate familial relationship between the two disorders. Further theorizing about the nature of this relationship needs to account for the much greater specificity of the parent–offspring transmission of BD compared to MD.

## Supporting information

10.1017/S0033291726103286.sm001 Kendler et al. supplementary material Kendler et al. supplementary material

## Data Availability

Kristina Sundquist, MD, PhD, had full access to all the data in the study and takes responsibility for the integrity of the data and the accuracy of the data analysis.

## Supplementary material

The supplementary material for this article can be found at http://doi.org/10.1017/S0033291726103286.
