# Supplementary material for: The risk for major depression and bipolar disorder in the offspring of informative parental mating types: a Swedish population-based study
Source: Psychol Med. 2026 Feb 11;56:e44. doi: 10.1017/S0033291726103286 (PMC12902172; doi:10.1017/S0033291726103286)
Supplement: Kendler et al. supplementary material [file S0033291726103286sup001.docx]

Appendix – The Risk for Major Depression and Bipolar Disorder in the Offspring of the Parental Mating Types of Major Depression and Bipolar Disorder: A Swedish Population-Based Study

Table 1 – Description of Registers

*Total Population Register*

The Total Population Register was created digitally in 1968 and includes yearly information, from the National Tax Board, on individuals registered in Sweden. It is possible to retrieve information on e.g. birth, death, immigration, emigration, migration within Sweden, place of residence, family information, civil status, etc. For more information, see [*https://www.scb.se/contentassets/8f66bcf5abc34d0b98afa4fcbfc0e060/rtb-bar-2016-eng.pdf*](https://www.scb.se/contentassets/8f66bcf5abc34d0b98afa4fcbfc0e060/rtb-bar-2016-eng.pdf)

*Multi-Generation Register*

The Multi-Generation Register is a register made up of persons who have been registered in Sweden at some time since 1961 and those who were born in 1932 or later. These are called index persons. The register contains connections between index persons and their biological parents. In 2024, more than 11 million index persons were included in the register. The Multi-Generation Register is a part of the register system for Total Population Register, where information comes from the National Tax Board. Every year, a new version of the register is created, including new index persons who immigrated or were born during the year. Information from the Multi-Generation Register may be disclosed for research and statistical purposes. See *https://www.scb.se/vara-tjanster/bestall-data-och-statistik/register/flergenerationsregistret/*

*National Patient Register*

In the 1960's the National Board of Health and Welfare started to collect information regarding in-patients at public hospitals, the National Patient Register (NPR). Initially it contained information about all patients treated in psychiatric care and approximately 16 percent of patients in somatic care. The register at that time covered six of the 26 county councils in Sweden. In 1984, the Ministry of Health and Welfare together with the Federation of County Councils decided a mandatory participation for all county councils. From 1987, NPR includes all in-patient care in Sweden. Since 2001, the register also covers outpatient doctor visits including day surgery and psychiatric care from both private and public caregivers. For more information, see *https://www.socialstyrelsen.se/en/statistics-and-data/registers/national-patient-register/*

*Primary Care Data*

We also used information from our new Primary Care research dataset including individual-level information on clinical diagnoses from primary health care centers from the following Swedish counties: Blekinge (2009-2018), Dalarna (2005-2018), Gotland (2011-2018), Gävleborg (2010-2018), Halland (2007-2018), Jönköping (2008-2018), Kalmar (2007-2018), Kronoberg (2006-2018), Norrbotten (2001-2018), Skåne (1989-2018), Stockholm (2003-2018), Södermanland (1992-2018), Uppsala (2005-2018), Västra Götaland (2000-2018), Värmland (2005-2018), Västerbotten (1991-2018), Västernorrland (2008-2018), Västmanland (2014-2018), Östergötland (1990-2018), and Örebro (2006-2018). The retrieval of data differs due to timing of digitalization of patient records. In 2018, 99% of the Swedish population lived in these 20 counties. For more information see *Sundquist, J., Ohlsson, H., Sundquist, K. et al. Common adult psychiatric disorders in Swedish primary care where most mental health patients are treated. BMC Psychiatry 17, 235 (2017).*

*The Population and Housing Censuses*

Every fifth year between 1960 and 1990 Sweden conducted censuses. These registers include among other things, the population's employment, the composition of households and housing. *For more information, see https://www.scb.se/en/finding-statistics/statistics-by-subject-area/population-and-living-conditions/population-composition-and-development/population-and-housing-census-1960-1990-tpr/*

Table 2 - Definition of Diagnoses

|  | Registers Used | Definition |
| --- | --- | --- |
| Bipolar Disorder  (BD) | Hospital Discharge Register;  Outpatient Care Register; Primary Care Data; | Bipolar Disorder (BD) was identified in the Swedish medical registries by ICD codes: ICD8: 296.1, 296.3, 296.8, 296.9, 298.1; ICD9: 296A, 296C, 296D, 296E, 296W, 298B; ICD10: F30 (excluding F30.0), F31 (excluding F31.0). |
| Major Depression  (MD) | Hospital Discharge Register;  Outpatient Care Register; Primary Care Data; | Major Depression (MD) was identified in the Swedish medical registries by ICD codes: ICD8: 296.0, 296.2, 298.0, 300.4; ICD9: 296B, 298A, 300E; ICD10: F32, F33. |
| Schizophrenia (SZ) | Hospital Discharge Register;  Outpatient Care Register; Primary Care Data; | Schizophrenia (SZ) was identified in the Swedish medical registries by ICD codes: ICD8: 295.1, 295.2, 295.3, 295.6, 295.9; ICD9: 295B, 295C, 295D, 295G, 295X; ICD10: F20.0, F20.1, F20.2, F20.3, F20.5, F20.9. |

Table 3 – Details on R-packages used in statistical analyses

1. Therneau T. survival: A package for Survival Analysis in R. R package. 2024.
2. Bonett D. statpsych: Statistical Methods for Psychologists. R package. 2024.
3. Wickham H, Miller E, Smith D. haven: Import and Export 'SPSS', 'Stata' and 'SAS' Files. R package. 2023.
4. Wickham H, François R, Henry L, Müller K, Vaughan D. dplyr: A Grammar of Data Manipulation. R package. 2023.
5. Barrett T, Dowle M, Srinivasan A, Gorecki J, Chirico M, Hocking T, Schwendinger B. data.table: Extension of ‘data.frame’. R package. 2024.
6. Viechtbauer W. Conducting meta-analyses in R with the metafor package. *J Stat Softw.* 2010;36(3):1-48.

Table 4 – Descriptive statistics of the study in terms of sample size, birth year, age, sex distributions, parental and offspring prevalences, Conversion rates of MD -> BD and ages at first registration of Bipolar Disorder (BD), and Major Depression (MD). Amongst broken families, with offspring having a recorded biological mother and biological father, with parental follow-up time available at least until 1976.

|  | All offspring | | Female offspring | | Male  offspring | | Biological mother | | Biological father | |
| --- | --- | --- | --- | --- | --- | --- | --- | --- | --- | --- |
| Number | 795,833 | | 394,478 | | 401,355 | | 795,833 | | 795,833 | |
|  | Mean | SD | Mean | SD | Mean | SD | Mean | SD | Mean | SD |
| Year of birth | 1980.3 | 6.5 | 1980.2 | 6.5 | 1980.4 | 6.4 | 1953.5 | 7.7 | 1950.4 | 8.4 |
| Age at follow-up | 37.0 | 7.3 | 37.0 | 7.4 | 37.0 | 7.2 | 63.6 | 8.5 | 64.9 | 9.6 |
| Prevalence rates, % | | | | | | | | | | |
| BD total | 2.02 | | 2.69 | | 1.38 | | 1.92 | | 1.21 | |
| MD only | 20.28 | | 25.49 | | 15.16 | | 24.23 | | 13.17 | |
| Conversion rates, MD -> BD | | | | | | | | | | |
| All with first MD, after total follow-up time (among those with at least 5 years of follow-up time available) | 8.40 | | 9.14 | | 7.07 | | 5.75 | | 5.49 | |
| Age at first diagnosis | | | | | | | | | | |
|  | Mean | SD | Mean | SD | Mean | SD | Mean | SD | Mean | SD |
| BD total | 30.5 | 6.9 | 30.4 | 6.9 | 30.8 | 6.9 | 48.6 | 11.1 | 50.2 | 11.9 |
| MD only | 30.2 | 7.2 | 30.1 | 7.3 | 30.2 | 7.1 | 52.0 | 11.2 | 54.1 | 12.4 |

Table 5 – Tetrachoric Correlations (TC) and Hazard Ratios (HR) of Mating Combinations of Parental Bipolar Disorder (BD) and Major Depression (MD) With Offspring BD and MD amongst broken families.

| **Tetrachoric Correlations** | | | | | | | | | | | |
| --- | --- | --- | --- | --- | --- | --- | --- | --- | --- | --- | --- |
|  | A. UN x UN | B. BD^-^**MD^+^** x UN | | C. **BD^+^**MD^-^ x UN | | D. BD^-^**MD^+^** x BD^-^**MD^+^** | | E. BD^-^**MD^+^** x **BD^+^**MD^-^ | | F. **BD^+^**MD^-^ x **BD^+^**MD^-^ | |
| Number of offspring | 508,910 | 231,923 | | 19,411 | | 30,390 | | 4,932 | | 267 | |
| Offspring diagnosis |  | TC | 95% CI^a^ | TC | 95% CI^a^ | TC | 95% CI^a^ | TC | 95% CI^a^ | TC | 95% CI^a^ |
| BD total | Ref | 0.12 | 0.11,0.13  **** | 0.40 | 0.39,0.42  **** | 0.24 | 0.22,0.26  **** | 0.48 | 0.45,0.50  **** | 0.65 | 0.59,0.70  **** |
| MD only | Ref | 0.16 | 0.15,0.16  **** | 0.13 | 0.12,0.14  **** | 0.25 | 0.24,0.25  **** | 0.20 | 0.19,0.22  **** | 0.19 | 0.11,0.26  **** |
| Conversion to BD^b^ | Ref | 0.03 | 0.01,0.04  *** | 0.27 | 0.25,0.30  **** | 0.06 | 0.03,0.08  **** | 0.29 | 0.25,0.33  **** | 0.43 | 0.32,0.53  **** |
| **Hazard Ratios** | | | | | | | | | | | |
| BD total | Ref | 1.53 | 1.50,1.56  **** | 4.05 | 3.93,4.16  **** | 2.19 | 2.12,2.27  **** | 5.48 | 5.28,5.69  **** | 11.74 | 10.66,12.94  **** |
| MD only | Ref | 1.50 | 1.49,1.51  **** | 1.49 | 1.48,1.51  **** | 2.07 | 2.06,2.08  **** | 1.93 | 1.91,1.96  **** | 1.84 | 1.74,1.94  **** |
| Conversion to BD^b^ | Ref | 1.04 | 1.00,1.09 | 2.15 | 1.98,2.34  **** | 1.10 | 1.01,1.19  * | 2.32 | 2.03,2.65  **** | 3.77 | 2.50,5.69  **** |
| ^a^ Significance levels for p-values: *<.05, **<.01, ***<.001, ****<.0001  ^b^All with MD onset first are included, having at least 5 years of follow-up after onset; n = 112,861 offspring. | | | | | | | | | | | |
